# Efficacy of dabrafenib/trametinib in pancreatic ductal adenocarcinoma with BRAF NVTAP deletion: A case report

**DOI:** 10.3389/fonc.2022.976450

**Published:** 2022-11-24

**Authors:** Ji Eun Shin, Ho Jung An, Hyung Soon Park, Hyunho Kim, Byoung Yong Shim

**Affiliations:** Division of Medical Oncology, Department of Internal Medicine, St. Vincent’s Hospital, Catholic University of Korea, Suwon-si, Gyeonggi-do, South Korea

**Keywords:** pancreatic ductal adenocarcinoma, BRAF mutation, BRAF short in-frame deletion, dabrafenib/trametinib, NGS

## Abstract

Studies have been actively conducted to identify actionable mutations and incorporate them into clinical practice in pancreatic ductal adenocarcinoma (PDAC), which is known to have a poor prognosis with traditional cytotoxic chemotherapy. A BRAF point mutation in V600E is commonly reported in KRAS wild-type PDAC, and targeting BRAF_V600E is already being applied to various carcinomas, including PDAC. Accumulated evidence also shows that not only BRAF_V600E but also short in-frame deletions of BRAF have an oncogenic function. Here, we report that a patient with BRAF N486_P490 deletion initiated on dabrafenib or trametinib, a BRAF inhibitor, and a MEK inhibitor, respectively, after cytotoxic chemotherapy failure. The patient then presented with a partial response.

## Introduction

PDAC is one of the most lethal diseases, with the 5-year survival rate remaining as low as 6% in the USA (1). A substantial number of patients are diagnosed at advanced stages without symptoms. Curative resection can be performed in only 20% of patients who are diagnosed at the early stages; still, most of them experience recurrence after surgical resection. Based on current evidence (2), cytotoxic chemotherapy is the mainstay of treatment for metastatic PDAC based on current evidence (2). However, the expected median overall survival period after standard cytotoxic chemotherapy is less than 12 months, and most patients suffer from progression, refractory to conventional therapies (3, 4).

Currently, sequencing of the genetic alterations in PDAC enables approaches for target therapies (5, 6). There are four known major driver genes for pancreatic cancer: KRAS, CDKN2A, TP53, and SMAD4 (7). However, these mutations have no amendable target agent in the clinical field to date. The target agents currently approved for PDAC are entrectinib/larotrectinib and olaparib for patients harboring NTRK fusion or BRCA 1/2 mutations (8–10).

BRAF, a member of the RAS–RAF–MEK–ERK (MAPK) cascade, is one of the most commonly mutated kinases in many carcinomas. The V600E mutation in BRAF is the most frequently detected in various carcinomas, and target agents such as vemurafenib and dabrafenib were developed (11). Dabrafenib and trametinib demonstrated high rates of survival in patients with a BRAF V600E mutation and were approved by the FDA for treating stage IV BRAF V600E-mutated non-small cell lung cancer, melanoma, and anaplastic thyroid cancer (12–14). Also, in PDAC, about 4% of patients harbor the BRAF mutation, which correlates inversely with KRAS (15), and results of clinical efficacy using the BRAF inhibitor/MEK inhibitor doublet in PDAC with the BRAF mutation have been reported recently (16–18).

In addition to V600E, other BRAF missense mutations have been identified recently. The N486_P490 deletion (NVTAP deletion), one of the BRAF short in-frame deletions, reveals oncogenic activity analogous to its V600E mutation in preclinical studies. As NVTAP deletion has a similar mode of action to BRAF_V600E, it appears reasonable to assume that NVTAP deletion may also be effectively treated with a previously developed BRAF inhibitor (19). A single case was previously reported where dabrafenib was used for NVTAP deletion in PDAC (20). The following case describes a patient who has PDAC with BRAF NVTAP deletion and is treated with dabrafenib or trametinib, a BRAF inhibitor or MEK inhibitor.

## Case presentation

An 83-year-old male presented with uncontrolled hyperglycemia and abdominal pain for a week. On the abdomen CT scan, a 3.5 × 2.5 cm sized pancreas body mass invading the common hepatic artery was noticed with intrahepatic, common bile duct dilatation, and small peritoneal nodules. Endoscopic ultrasound-guided fine needle aspiration for pancreatic body mass was conducted, and cytologic diagnosis showed a few clusters of atypical cells, resulting in the diagnosis of adenocarcinoma. Peritoneal carcinomatosis was found in the PET CT. After an overall examination, he was diagnosed with PDAC, cT4N1M1, stage IV. The patient received two cycles of cytotoxic chemotherapy: gemcitabine and albumin-bound paclitaxel. His disease continued to progress, as evidenced by the increasing size of the pancreatic body mass (3.6 × 3.0 cm) and the increased amount of ascites, thereby being determined as a progressive disease according to RECIST 1.1 criteria. Although mFOLFIRINOX could have been used as second-line palliative chemotherapy in the country, the patient’s condition would have been intolerable considering his performance status.

**Figure 1:**
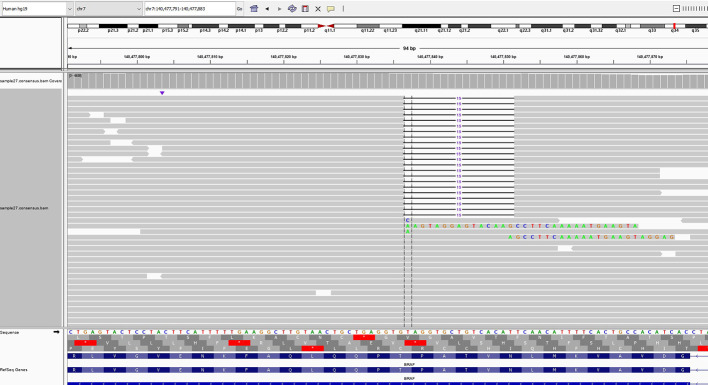
Next-generation sequencing of the liquid biopsy specimen revealed a NVTAP (N486_P490) deletion. The data was provided by the Department of Research and Development, Foundation Medicine Inc.

Tumor molecular profiling using next-generation sequencing (FoundationOne Liquid CDx) was performed through liquid biopsy with a blood sample from the patient. The patient exhibited microsatellite stability, and the blood tumor mutational burden was 0 Muts/Mb. The genomic findings revealed the mutations of BRAF NVTAP deletion (**Figure 1**), PTEN G127R, CDKN2A/B p16INK4a V25fs*12, MUTYH splice site 892-2A>G, and TP53 R248Q. **Table 1** summarizes the tier according to ESCAT and VAF each genomic alteration. As mentioned above, BRAF NVTAP deletion is reported as an oncogenic mutation in PDAC. There was a single previous case where dabrafenib was used to target BRAF NVTAP deletion, and the clinical effectiveness of dabrafenib was demonstrated in this mutant (20). We requested and obtained dabrafenib/trametinib from Novartis with the Managed Access Program.

**Table 1:** Genomic findings of next-generation sequencing (FoundationOne Liquid CDx).

Gene		VAF (%)	ESCAT
BRAF	N486_P490del	0.30	II
PTEN	G127R	0.42	IV
CDKN2A/B	p161NK4a V25fs*12	0.21	IV
MUTYH	Splice site 892-2A>G	49.6	X
TP53	R248Q	0.32	IV

**Figure 2:**
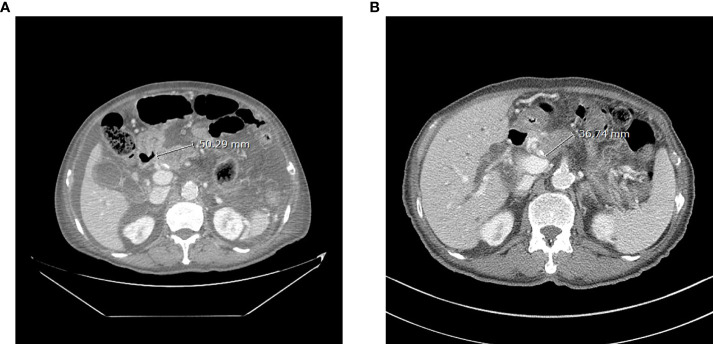
Abdominal CT of the patient before (**A**, 7 July 2021) and after (**B**, 6 September 2021) 8 weeks of dabrafenib use.

After receiving dabrafenib 150 mg twice a day and trametinib 2 mg once a day for 8 weeks, a radiologic evaluation showed a marked decrease in primary pancreas body mass (50 mm to 36 mm) with improved ascites (**Figure 2**). Findings were rated as a partial response according to RECIST 1.1 criteria, and it was maintained for 6 months with continued treatment. The toxicities of drugs such as pyrexia, headache, diarrhea, and rash were not noticed. However, unfortunately, the patient died of an outbreak of SARS-CoV-2 infection during treatment.

## Discussion

While the most frequent oncogenic BRAF mutation is V600E, emerging evidence shows that BRAF in-frame deletions also have oncogenic activity. Moreover, it revealed that BRAF NVTAP deletion affects BRAF dimer formation in human cancers. The deletion of five amino acids (NVTAP) in the kinase domain (b3-aC loop) constrains aC from an “out” to an “in” conformation, which is an active formation of BRAF kinase. This conformational change leads BRAF to autonomous kinases (19).

As BRAF inhibitors selectively bind to the ATP-binding site of BRAF_V600E kinase, attempts were made to identify whether NVTAP deletion is also affected by developed BRAF inhibitors. In cell line analysis, the NVTAP deletion mutant is sensitive to dabrafenib but resistant to vemurafenib. The geometric change of NVTAP deletion, a shortened b3-aC loop leading to kinase “in” conformation, makes vemurafenib resistant because it usually binds aC “out” conformation. However, dabrafenib, another “out” inhibitor, causes a lesser aC shift than that of vemurafenib, which results in minimal steric clash, and therefore the efficacy of this inhibitor can be maintained (4, 21).

KRAS wild-type PDAC accounts for 10% of total patients and represents a distinct molecular subtype with the possibility of the efficacy of the target agent (18). It can be classified as tumors with an activated alternative MAPK pathway, tumors with microsatellite instability, and tumors with kinase fusion genes (22). Interestingly, KRAS wild-type patients were significantly accompanied by mutations in BRAF, CTNNB1, ERBB2, MET, KIT, NTRK1, and FGFR4, which may be primarily associated with the activation of the RTK/RAS/MAPK pathway (23). The BRAF mutation is generally mutually exclusive with the KRAS mutation and occurs in 30% of KRAS wild-type PDAC cases (24). Notably, the proportion of BRAF deletion among BRAF mutations in PDAC is higher than in other carcinomas where the V600E mutation is relatively common. Genomic analysis revealed BRAF in-frame insertions or deletions in approximately 10% of patients with KRAS wild-type PDAC or 1% of all PDAC patients (25).

Because BRAF NVTAP deletion plays an oncogenic role in PDAC, this case demonstrates that BRAF in-frame deletion is targetable and can be treated with known BRAF inhibitors that were previously described in cell line assays. It suggests that targeted therapy through individual molecular studies can be a new treatment direction for PDAC patients with poor prognoses.

## Conclusion

We described a case of PDAC harboring a BRAF NVTAP in-frame deletion in a patient with a partial response to target therapy with dabrafenib and trametinib. It was an opportunity to examine the effectiveness of known target therapy for the rare mutation of BRAF, and we suggest that personalized medicine can be a further direction of treatment for tumor patients with poor prognoses.

## Data availability statement

The datasets presented in this study can be found in online repositories. The names of the repository/repositories and accession number(s) can be found in the article/supplementary material.

## Ethics statement

Written informed consent was obtained from the individual(s) for the publication of any potentially identifiable images or data included in this article.

## Author contributions

JS and BS had full access to data in this case and wrote the manuscript. HA, HP, and HK reviewed the written manuscript and participated in the modification. All authors contributed to the article and approved the submitted version.

## Acknowledgments

The data presented here are the result of a valuable collaborative process among the medical oncology department staff at St. Vincent Hospital, Republic of Korea. Drugs were supported by the MAP program from Norvatis.

## Conflict of interest

The authors declare that the research was conducted in the absence of any commercial or financial relationships that could be construed as a potential conflict of interest.

## Publisher’s note

All claims expressed in this article are solely those of the authors and do not necessarily represent those of their affiliated organizations, or those of the publisher, the editors and the reviewers. Any product that may be evaluated in this article, or claim that may be made by its manufacturer, is not guaranteed or endorsed by the publisher.
